# Recurrence of acute myeloid leukemia in cryptorchid testis: case report

**DOI:** 10.1590/S1679-45082014RC2689

**Published:** 2014

**Authors:** Luccas Santos Patto de Góes, Roberto Iglesias Lopes, Octavio Henrique Arcos Campos, Luiz Carlos Neves de Oliveira, Alexandre Crippa Sant'Anna, Marcos Francisco Dall'Oglio, Miguel Srougi

**Affiliations:** 1 Hospital do Servidor Público Municipal de São Paulo, São Paulo, SP, Brazil.; 2 Hospital das Clínicas, Faculdade de Medicina, Universidade de São Paulo, São Paulo, SP, Brazil.

**Keywords:** Leukemia, acute myeloid, Cryptorchidism, Orchiectomy, Testicular neoplasms, Recurrence, Chemotherapy, adjuvant, Case reports

## Abstract

A 23-year-old male with a history of bone marrow transplant for acute myeloid leukemia. He presented a large mass in the right inguinal region 5 years ago. Upon physical examination, right-sided cryptorchidism was observed. The tumor markers alpha-fetoprotein and beta-HCG were within normalcy range and lactate dehydrogenase was raised. Computed tomography of the abdomen and pelvis revealed right testicular mass in contiguity with the inguinal canal to the ipsilateral retroperitoneum, associated with right hydronephrosis. Due to the risk of germ-cell tumor in undescended testicle, the patient underwent radical right orchiectomy. The pathological examination showed recurrence of acute myeloid leukemia in the testis. He was referred to oncology for adjuvant therapy. Our literature review found no similar cases described.

## RESUMO

Paciente de 23 anos, masculino, com antecedente de transplante de medula óssea por leucemia mieloide aguda. Há 5 anos, apresentou volumosa massa em região inguinal direita. No exame físico, foi constatada criptorquidia à direita. Os marcadores tumorais alfafetoproteína e beta-HCG encontravam-se dentro da normalidade, e a desidrogenase láctica estava aumentada. A tomografia computadorizada de abdomen e pelve revelou massa testicular direita com contiguidade pelo canal inguinal, até o retroperitônio ipsilateral, associada a hidronefrose direita. Devido ao alto risco de neoplasia germinativa em testículo criptorquídico, o paciente foi submetido à orquiectomia radical direita, cujo anatomopatológico revelou recidiva de leucemia mieloide aguda em testículo. Foi encaminhado para oncologia para terapia adjuvante. Nossa revisão não revelou nenhum caso semelhante na literatura.

## INTRODUCTION

Acute myeloid leukemia (AML) is a myeloproliferative syndrome and accounts for 20% of acute leukemias in childhood, and for 50% in patients aged up to 20 years, mainly white males. Treatment is based on bone marrow transplant and chemotherapy.^(1)^

Cryptorchidism is the absence of testis in the scrotum and is one of the most common disorders in childhood, affecting 0.8 to 1% of young adults. Its diagnosis and treatment with orchidopexy must be made early, since the condition is associated to testicular torsion, hernias, infertility and malignization. The risk of degeneration and developing a germ-cell tumor is 40-fold higher as compared to the general population.^(2,3)^

Approximately 4% of pediatric patients with lymphoma may have a primary testicular involvement or relapse. The testis is considered to have a vascular barrier that hinders chemotherapeutical action, and orchiectomy is the standard treatment.^(4–6)^

The objective was to report a case of unidentified testicular mass in a clinical evaluation before bone marrow transplant and difficulty in making diagnosis and treat this condition. Based on our literature review, this might be the only case reported of AML recurrence in cryptorchidism.

## CASE REPORT

**Figure 1:**
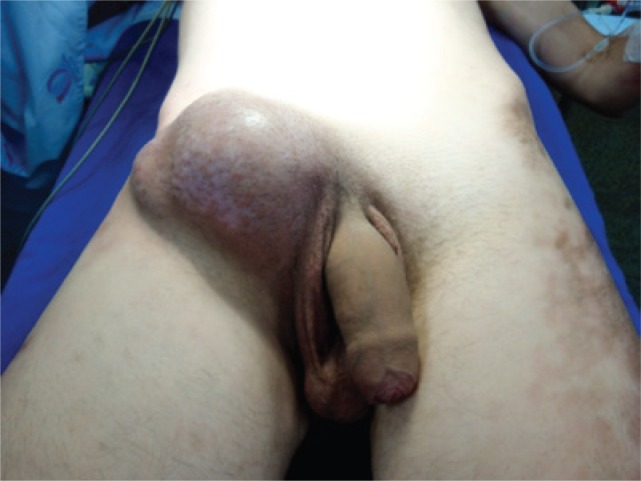
Right inguinal mass, hard and fixed to adjacent tissues

A 23-year-old male patient diagnosed as AML in 2003. He was submitted to allogeneic bone marrow transplant (donor: sister) in February 2004. One hundred days later he developed graft *versus* host disease presenting diffuse sclerodermiform plaques. He was treated with pulse therapy, and progressed with partial regression of the lesions. Five years later, a hard bulky mass, fixed to adjacent tissues, was observed in the right inguinal region (Figure 1). On physical examination, the ipsilateral testis was not identified.

**Figure 2:**
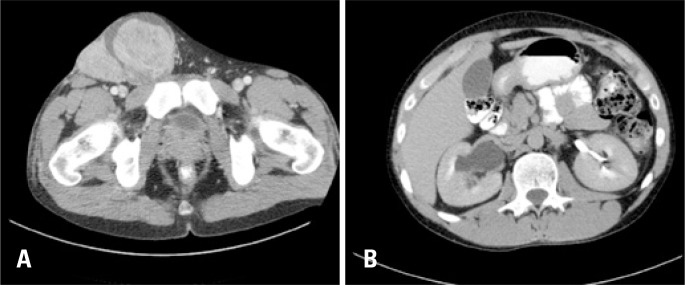
(A) Computed tomography showed a right testicular mass in contiguity with the ipsilateral inguinal canal; (B) hydronephrosis on the right due to compression by the scrotal mass that extended to the retroperitoneum

The hypothesis of testicular cancer associated to right cryptorchidism was raised. The tumor markers were alpha fetoprotein: 2.5ng/mL (reference value (RV): up to 7ng/mL), beta-HCG: normal (RV: <3U/mL) and lactate dehydrogenase: increased 483UI/L (100 a 190UI/L). Computed tomography (CT) of abdomen and pelvis showed a right testicular mass with contiguity to the inguinal canal up to the ipsilateral retroperitoneum, associated to right hydronephrosis (Figures 2A and 2B).

**Figure 3:**
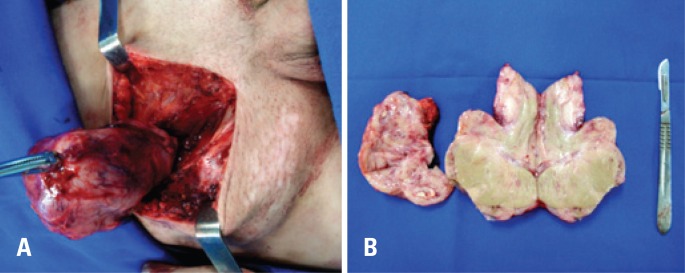
(A) Right radical orchiectomy of an inguinal mass related to a cryptorchid testis tumor. (B) Testicular mass and peritesticular lymph node after orchiectomy

**Figure 4:**
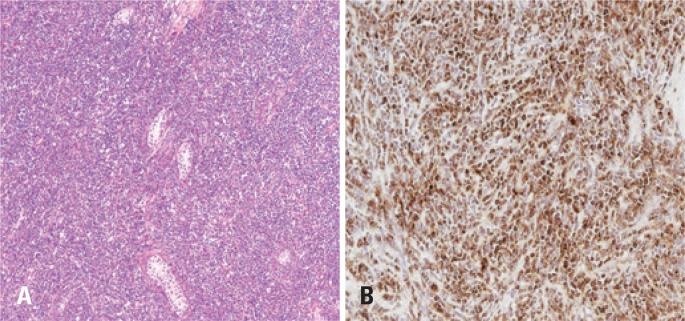
(A) Histology demonstrating anarchic cell hyperproliferation suggestive of leukemia. (B) Immunohistochemistry for myeloperoxidase revealed acute myeloid leukemia

Right radical orchiectomy was indicated due to suspected primary tumor or recurrence of AML. Inguinotomy was performed and an enlarged and hard peritesticular lymph node was excised (Figures 3A and 3B). The frozen biopsy revealed epithelial-cell lineage with high mitotic index, but with no defined histological pattern. The pathological report was diffuse infiltration by AML in testis and peritesticular lymph node (Figures 4A and 4B). The patient had no postoperative intercurrent events, and was discharged two days after surgery. He was referred to oncology for adjuvant therapy.

## DISCUSSION

Testicular cancer is frequent in young adult population, with an annual incidence of 4 per 100 thousand, and 95% are germ-cell tumors.^(7,8)^ In this case reported, a rare type of neoplasm is observed and it corresponds to less than 0.5% of testicular tumors, caused by infiltration of leukemia/lymphoma of the testis. The most frequent subtype in testis is lymphoid leukemia. In the young adult population with acute lymphoid leukemia, up to 5% of patients can have primary involvement or relapse in testis.^(6)^ There are case reports in the literature of recurrent acute lymphoid leukemia after bone marrow transplant, in whom chemotherapy and radiation therapy were not efficient and orchiectomy was the treatment chosen for remission of the disease.^(9,10)^

Testicular involvement is extremely rare in AML, and there are few cases reported, mainly in children; in that, bone marrow involvement occurred later in almost all patients. In these cases, the interval between remission and testicular recurrence ranged from 4 to 60 months, in average 21 months.^(4)^ In our patient, the interval was of 60 months. Several studies stated that testicular involvement in leukemias must be treated with orchiectomy due to the vascular barrier that hinders the action of chemotherapy.^(4–6)^

In this present case orchiectomy was indicated because of undefined testicular tumor, since the tumor markers did not suggest the diagnosis. Computed tomography of abdomen and pelvis is mandatory in cases of testicular tumors for staging and searching primarily retroperitoneal adenomegaly, for these lymph node basins are more affected in such neoplasms.^(7)^ The intraoperative frozen biopsy was performed to verify the presence of malignant lesion. The literature shows that relapsed AML or primary tumor of the testis should be addressed by orchiectomy, and the treatment complemented with adjuvant chemotherapy, based on the pathological examination.^(4–6)^ The case was managed according to the current recommendations, considering the peculiar recurrence of AML, a rare event that affected a patient with cryptorchidism, which hindered making diagnosis.
